# Chronic Traumatic Encephalopathy: A Brief Overview

**DOI:** 10.3389/fneur.2019.00713

**Published:** 2019-07-03

**Authors:** Arman Fesharaki-Zadeh

**Affiliations:** Yale Neurology, Yale Medicine, New Haven, CT, United States

**Keywords:** TBI, CTE, tau & phospho-tau protein, concussion and sports, dementia pugilistica

## Abstract

Chronic Traumatic Encephalopathy (CTE) is a debilitating neurodegenerative disease, which has been increasingly reported in athletes, especially American football players, as well as military veterans in combat settings, commonly as a result of repetitive mild traumatic brain injuries (TBIs). CTE has a unique neuropathological signature comprised of accumulation of phosphorylated tau (p-tau) in sulci and peri-vascular regions, microgliosis, and astrocytosis. As per most recent disease classification, the disease manifests itself in four different stages, characterized by widespread tauopathy. Clinically, CTE has a more subtle presentation, as patients often present with two distinct phenotypes, with one subtype initially presenting with affective changes, and the other subtype with more cognitive impairment. On a genetic basis, there are no clear risk factor genes. Although ApoE4 carriers have been reported to suffer more severe outcome post TBI. As there are no disease modifying regimen for CTE, the newly developed TBI treatments, if administered in a time sensitive manner, can offer a potential viable option. Prevention is another key strategy that needs to be implemented in various sports and military settings. Providing education for safe practice techniques, such as safe tackling and hitting, and providing ready access to full neuropsychiatric assessment by team physician could have measurable benefits. The combination of advanced of research techniques including neuroimaging, as well as increasing public awareness of CTE, offers promising vistas for research advancement.

Chronic Traumatic Encephalopathy (CTE) is a distinctive tau-protein associated neurodegenerative disease. There has been a rise of CTE diagnosis in athletes, especially American football players, as well as in military veterans in combat settings (1, 2). Although CTE has been publicly recognized relatively recently, it was first described as “punch drunk” syndrome in a classic article by Martland et al. (3). The report was focused on a number of boxers who had suffered repetitive head blows throughout their careers, and were presenting with both psychiatric symptoms as well as severe memory and neurocognitive deficits, analogous to typical dementia patients (3). The disease nomenclature evolved into “dementia pugilistica” (4), and finally CTE in 1949 (5).

CTE has a unique neuropathological characteristic, comprised of accumulation of phosphorylated tau (p-tau) in sulci and peri-vascular regions, microgliosis, and astrocytosis. These pathological changes lead to progressive debilitating neurodegeneration. Based on the pattern of pathological progression, CTE is divided into four respective stages (Figure 1). In stage I CTE, the brain grossly appears normal, but p-tau is found in a finite number of loci, often in the lateral and frontal cortices, as well as proximal to small blood vessels in the depth of sulci. There might be a scant number of neurofibrillary tangles (NFTs) and neurites in the locus coeruleus. In stage II, localized macroscopic abnormalities might be noted. On gross anatomical sections and neuroimaging, enlargement of lateral ventricles, cavum septum pellucidum with or without fenestration, as well as pallor of the locus coeruleus and substantia nigra are observed. There are multiple foci of p-tau within the depth of sulci, and there is an emergent spreading pattern. In stage III, most gross pathological sections show macroscopic abnormalities. There is global brain weight loss, mild frontal lobe and temporal lobe atrophy, and dilation of the ventricles. One half of CTE patients display septal abnormalities, including cavum septum pellucidum. P-tau pathology spreads, involving the frontal, temporal, parietal and insular cortices. In stage IV, the reduction in brain weight is dramatic, and brain weights of 1,000 g (compared to 1,300–1,400 g in normal brains) have been reported. There is profound atrophy of the frontal, medial temporal lobes, as well as anterior thalami. There is also atrophy of the white matter tracts. The majority of stage four patients have septal abnormalities. The spread of the p-tau affects most regions, including the calcarine cortex (7, 8). Abnormalities in phosphorylated 43 kDa TAR DNA binding protein (TDP-43) is also seen in most CTE patients. The parenchymal TDP-43 pathology is also progressive in nature similar to the anatomical pattern of spread of p-tau. TDP-43 immunoreactivity is found in almost all cases of stage IV disease (7).

**Figure 1:**
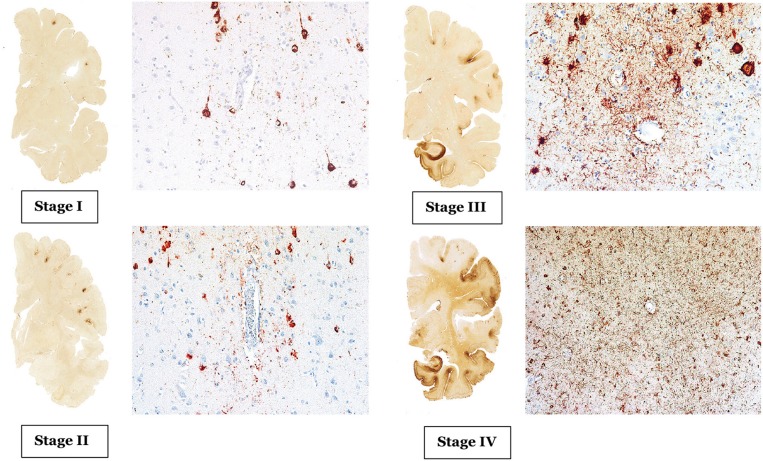
The above images are depiction of the McKee's four stages of CTE [adopted from Mez et al. (6), Figures 1, 2].

The CTE clinical phenotype is yet to be clearly defined. The following paragraphs outline attempts of characterization of CTE symptoms in the various stages of the disease process (Table 1). According to McKee's classification, in stage I, a typical CTE patient is asymptomatic, or may complain of mild short term memory deficits and depressive symptoms. Mild aggression may be observed. In Stage II, the mood and behavioral symptoms could include behavioral outbursts and more severe depressive symptoms. In Stage III, patients typically present with more cognitive deficits, including memory loss, executive functioning deficits, visuospatial dysfunction, and apathy. In Stage IV, patients present with advanced language deficits, psychotic symptoms including paranoia, motor deficits, and parkinsonism.

**Table 1:** CTE proposed clinical classifications.

**Classification**	**Diagnostic subgroups**	**Definition**
McKee et al. (9)	Stage I	• Asymptomatic, or mild memory and depressive symptoms.
	Stage II	• Symptoms include behavioral outbursts and severe depression
	Stage III	• Cognitive deficits including memory loss and executive dysfunction
	Stage IV	• Advance language deficits, psychotic symptoms, profound cognitive deficits, and motor features.
Jordan et al. (10)	Definite CTE	• Clinical CTE symptoms with supportive neuropathology
	Probable CTE	• Two or more CTE clinical symptoms consistent with CTE
	Possible CTE	• Consistent with CTE or other neurodegenerative diagnosis such as AD, PD, ALS
	Improbable CTE	• Not consistent with CTE symptoms examples include MS, or brain tumors
Stern et al. (11)	Behavioral CTE subgroup	• Initial presentation of mainly mood/behavioral symptoms
	Cognitive CTE subgroup	• Initial presentation of mainly cognitive impairment
Gardner et al. (12)	Classic CTE	• Initial presentation typically includes parkinsonism with later progression to cognitive symptoms
	Modern CTE	• Early clinical symptoms include mood/affective symptoms with later progression to cognitive symptoms
Montenigro et al. (13)	Traumatic encephalopathy syndrome (TES) l. TES behavioral/mood variant ll. TES cognitive variant lll. TES mixed variant lV. TES dementia	• Based on core clinical features including cognitive, behavioral, and mood domains • Supportive features including impulsivity, anxiety, apathy, paranoia, suicidality, headache, motor signs, documented functional decline, and delayed onset of symptoms for at least 2 years after significant head impact exposure
	Additional CTE based classification: l. Probable CTE ll. Possible CTE lll. Unlikely CTE ^*^CTE Biomarkers: (1) Cavum septum pellucidum (2) Normal amyloid-beta CFS level, as opposed to CSF amyloid beta elevation in AD (3) Elevated p-tau/total tau ratio compared to age-matched controls (4) Positive Tau neuroimaging such as Tau-PET imaging (5) Negative amyloid imaging, such as amyloid PET Brain scan, in order to delineate from possible AD (6) Cortical thinning (7) Cortical atrophy	• Probable CTE group has at least one positive CTE biomarker such Tau PET imaging vs. possible have progressive CTE course with any biomarker testing vs. in the unlikely CTE group, TES diagnosis not satisfied or negative Tau imaging or both • If the clinical presentation also included motor signs such as parkinsonism, the modifier “with motor features” was also added

*Summary of clinical stages of CTE according to various proposed classifications*.

Jordan et al. (10) were one of the first to clinically characterize the disease. They divided CTE clinical presentations into three domains: behavioral/psychiatric, cognitive, and motor. The behavioral and psychiatric domain included aggression, depression, apathy, impulsivity, delusions including paranoia, and suicidality. The cognitive domain included diminished attention and concentration, memory deficits, executive functioning deficits, visuospatial dysfunction, language deficits, and dementia. Finally, the motor features consisted of dysarthria, gait abnormalities, ataxia and incoordination, spasticity, and parkinsonism features such as tremors. Based on these clinical features, as well as existent neuropathological information, four diagnostic subtypes were defined, namely “Definite,” Probable,” “Possible,” and “Improbable” CTE.

Stern et al. (11), and related case reports (14, 15), differed in their description of a typical CTE patient, conceptualizing the clinical presentation into two distinct subtypes. The first subtype displayed mainly behavioral and mood changes, and the other presented with mainly cognitive impairment. The vast majority of the mood/behavior subtype developed cognitive deficits as the disease progressed. However, relatively few patients of the cognitive group displayed mood or behavior alterations during the course of their illness. In study by Stern et al. (11), the cognitive group patients had a significantly higher probability of developing dementia. They also were significantly older at the time of diagnosis compared to the mood/behavior group patients. The behavioral subgroup of CTE patients can resemble patients suffering from behavioral variant frontotemporal dementia (bvFTD), which makes the clinical diagnosis more challenging. However, typical bvFTD characteristic behavioral manifestations such as apathy and disinhibition are often not seen in CTE patients (11, 16). Given the inherent heterogeneity of bvFTD, as well as similar tauopathic nature of both diseases, distinguishing bvFTD and CTE poses a diagnostic challenge.

Out of the behavioral symptoms of CTE, the association between suicide and CTE remains a topic under scrutiny in the literature. Earlier studies, such as the series of five professional athletes with a confirmed diagnosis of CTE reported by Omalu et al. (17), had suggested a strong relationship between CTE and suicide. The authors further suggested that the etiology of suicidal/parasuicidal behavior in the CTE population might be partly due to tauopathy in the form of neurofibrillary tangles and neuritic threads in strategic limbic brain nuclei such as locus ceruleus. Maroon et al. (18), reviewed 153 pathologically confirmed cases of CTE published between 1954 and 2013. They reported the suicide prevalence in the CTE population and accidental deaths to be 11.7 and 17.5%, significantly higher than the general population levels of 1.5 and 4.8%, respectively (18). Proponents of opposing view suggest that suicides have been mostly reported in earlier stages of CTE, and the association between disease progression and suicide remains unclear at this time (19).

In a meta-analysis of 158 case studies by Gardner et al. (12), CTE clinical symptoms were divided into “classic” vs. “modern” CTE symptoms, to draw a distinction between an older description of CTE cases centered mostly on boxers compared to a more evolved clinical description which also applies to professional American football players. Whereas, the “classic” CTE symptoms typically included dysarthria, movement difficulties, and later progression to memory deficits, the “modern” CTE picture also included neuropsychiatric symptoms, such as depressive symptoms, paranoia, social withdrawal and isolation, compromised judgment and aggression. Cognitive deficits such as memory decline, executive dysfunction, language, and information processing deficits emerge later in the course of the disease process (12).

Since the definition of CTE primarily depends on pathological characteristics, there is a proposed alternative clinical term of traumatic encephalopathy syndrome (TES) by Montenigro et al. (13), describing the clinical sequelae of repetitive TBIs. The authors based this classification on a review of 202 published cases. TES is a more encompassing diagnosis and can be subdivided into four subcategories, including TES behavioral/mood variant, TES cognitive variant, TES mixed variant, and TES dementia. The proposed TES diagnosis was based on the existence of five general criterion, three core clinical features, and nine supportive features. Using existent biomarkers^*^ (Table 1), additional diagnostic qualifiers were proposed, which included “Probable,” “Possible,” and “Unlikely” CTE (9, 13). The proposed TES diagnosis also contained temporal qualifiers and included “progressive course,” “stable course,” and “unknown/inconsistent course.” If the clinical presentation also included motor signs such as parkinsonism, the modifier “with motor features” was also added.

As our understanding of CTE grows, there are a number of challenges and critiques that need to be addressed. One hypothesis as an alternative to the phenomena CTE, is a diminished “cognitive reserve” theory. The theory states that repetitive neurotrauma leads to a reduction in cognitive reserve and acceleration of development of an underlying neurodegenerative disorders (20, 21). If this theory held true, it would imply that CTE and AD are on the same neuropathological spectrum. This assertion deserves further analysis. Similar to AD, the Tau isoforms in CTE also consist of the mix of three-repeat (3R) and four-repeat (4R) isoforms. However, according to a recent report by Falcon et al. (22), the tau filaments extracted from the brains of CTE patients also contain a unique ß-helix region with a hydrophobic cavity, which is not present in the brains of AD patients. The cavity contains an additional cofactor that is thought to play a functional role in tau propagation. Falcon et al. (22) suggest that the location of tau inclusions in proximity to blood vessels, suggest that cofactors necessary for tau assembly may cross the blood brain barrier after head trauma. The authors further argue that the fact that brain trauma leads to CTE in only a subgroup of injured population, might be related to higher level of cofactors in the more susceptible individuals. These cofactors might provide a therapeutic target for prevention of tau assembly and development of CTE post injury (22).

An alternative theory proposes that the psychiatric symptoms such as depression and anger reported in CTE patients are independent of the CTE disease process and are reported in a cofounded fashion. The proponents of this hypothesis have cited prior studies such as the one reported by Weir et al. (23), in which 1,063 former NFL players were asked whether they have experienced bouts of anger. It was reported that 30.7% of the players ages 30–49, and 29.3% of the players ages 50 or above reported bouts of anger. However, the authors also noted that the reported measures of anger was indeed lower than the one reported for the general US population, which was 54.8% for men between 30 and 49, and 47.2% for men above the age of 50 (23). Though the arguments pertaining to comorbidity of psychiatric symptoms and neurodegenerative diseases such as CTE, are difficult to verify based on neuroimaging and neuropathological findings, one can apply similar arguments to psychiatric symptoms of any neurodegenerative condition such as AD, bvFTD, Parkinson Disease (PD) or amyotrophic lateral sclerosis (ALS).

Another important source of diagnostic confusion is the clinical delineation between CTE and prolonged post-concussive syndrome (PCS), especially given prior reports indicating that ~10–20% of individuals who suffer concussions, experience prolonged symptoms. Chronic Postconcussive Syndrome (CPCS) refers to persistence of PCS symptoms leading to impaired functional and often athletic performance lasting longer than 1 year. CPCS symptoms include headache, dizziness, impaired attention, memory and executive functioning deficits, depression and irritability symptoms (10). King and Kirkwilliam coined the term, “Permanent PCS” to refer to those with PCS symptoms persisting an average of 6.9 years after the initial concussion. Furthermore, they reported that a significant number of permanent PCS patients (40–59%) also had premorbid or postmorbid neuropsychiatric conditions such as depression, anxiety, PTSD, and/or pain (24). As argued by Jordan et al. (10), CPCS is clinically distinguishable from CTE, based on its temporal relationship to the acute concussive event. A thorough and accurate temporal history remains key in the neurological assessment. Furthermore, headache is a central feature of CPCS but not commonly reported in CTE. Although arguable, McKee stages I and II patients could present with headaches, further adding the complexity of possible overlap of CTE & CPCS (9). The CPCS diagnosis remains controversial, as it is not clear whether it is tauopathic in nature. Hence, the dividing lines of CPCS and McKee's stages I and II clinical characteristics are not fully solidified.

Clear genetic predispositions to CTE have not been reported. However, the ApoE4 gene, the most well-known risk factor for Alzheimer's Disease (25), has been associated with greater cognitive deficits and a more protracted recovery period after Traumatic Brain Injury (TBI) (11). A study on a group of boxers has reported more severe outcomes in individuals carrying at least one ApoE4 allele (26). Conversely, ApoE3 might confer neuroprotection, even in the presence of a progressive CTE pathology (15). Another proposed protective factor associated with more favorable post TBI recovery is cognitive reserve, as measured by premorbid IQ and total intracranial volume (27). Other genetic candidates for further study include the microtubule-associated protein tau (MAPT) gene, the progranulin (GRN) gene, and the chromosome nine open reading frame 72 (C9ORF72) gene (11).

The pathologic synergism of tauopathy and neuroinflammation is increasingly being recognized. Extracellular secretion of hyperphosphorylated tau is thought to activate microglia and astrocytes, leading to production of pro-inflammatory cytokines such as IL1ß, and TNFa, in turn leading to activation of tau kinases such as p38 and cdk5, and further tau phosphorylation. This process creates a vicious perpetual tauopathy and neuroinflammaion cycle (28). Given the robust association between repetitive traumatic brain injuries and risk of CTE (1), timely treatment of TBI could diminish the development of CTE. The pro-inflammatory nature of TBI has been previously reported (13), and anti-inflammatory agents such as minocycline with N-Acetylcysteine, a potent anti-oxidant, administered in acute to subacute time windows post TBI, offer a promising therapeutic regimen (29, 30). The development of a time sensitive protocol, resembling the treatment algorithm for ischemic stroke, would potentially measure long term outcome in post TBI recovery and prevention of development of subsequent CTE pathology (29).

There are currently no disease modifying medications for CTE, making prevention the most effective way of combating this debilitating neurodegenerative disease (31). Given the frequency of head collisions in contact sports such as American football, prevention of head trauma will require a cultural shift in the way the sport is taught and practiced. Training for safe practice techniques, such as safe tackling and hitting, while penalizing reckless hits will offer measurable benefits. Further changes must include creating an environment of safety, in which players are encouraged to report symptoms to referees, coaches as well as to team physicians. Furthermore, establishing a baseline neurocognitive profile could be used as a clinical reference marker to track changes in players' neuropsychiatric presentation. It is incumbent upon the team physicians to remove players from the field who have suffered even a mild uncomplicated TBI for further assessment (32).

There are a number of existent CTE related challenges to address. Although the incidence of sport related concussion has been reported to range from 1.6 million to 3.8 million, the incidence and prevalence of CTE remains largely unknown (33). One explanation for this lapse of knowledge is perhaps due to the fact that athletes exposed to cumulative subconcussive hits, which exert sufficient force to confer neuronal damage but initially have no overt clinical symptoms, are often not assessed or diagnosed in a timely manner (34). Large scale prospective studies, such as tracking athletes with multiple TBIs over a predefined period, would add to our understanding of the natural course and phenomenology of the disease. CTE is increasingly reaching the public spotlight via the mass media. Continuous efforts to diagnose, assess, and treat this devastating illness are needed. The exponential advancement in neuroimaging techniques and understanding of the neuropathological mechanisms of the illness will lead to earlier diagnosis and timely treatment interventions.

## Author Contributions

The author confirms being the sole contributor of this work and has approved it for publication.

### Conflict of Interest Statement

The author declares that the research was conducted in the absence of any commercial or financial relationships that could be construed as a potential conflict of interest.
